# Covariate-adjusted AP-1 motif architecture and footprinting track olaparib-adaptive chromatin remodelling

**DOI:** 10.3389/fsysb.2026.1859256

**Published:** 2026-09-09

**Authors:** Subhajit Dutta

**Affiliations:** Department of Biochemistry and Molecular Cell Biology (IBMZ), Center for Experimental Medicine, University Medical Center Hamburg-Eppendorf, Hamburg, Germany

**Keywords:** AP-1, ATAC-seq, chromatin accessibility, covariate adjustment, digital genomic footprinting, motif enrichment, olaparib adaptation, ovarian cancer

## Abstract

Transcription-factor involvement in drug-adaptive chromatin remodelling is often inferred from motif enrichment in differential ATAC-seq peaks, but such analyses can be distorted by sequence composition, peak length, baseline accessibility and redundant motif models. We reanalysed 105,048 consensus ATAC-seq peaks from an olaparib-adapted Kuramochi ovarian-cancer continuum using HOMER-calibrated AP-1/bZIP models with coordinate-level de-duplication. At T320, 27,154 peaks gained and 30,050 lost accessibility. AP-1 motif-occurrence density remained higher in gained chromatin under complementary adjustments: an all-peak spline-adjusted Poisson model gave a rate ratio of 1.90 (95% CI 1.80–2.01), overlap weighting gave 1.95 (1.82–2.11), and 14,046 covariate-matched pairs gave 1.97 (1.90–2.05). Following 35,325 fixed motif-positive/motif-negative peak pairs across the adapted series showed a positive accessibility trend of 0.0363 contrast units per dose doubling (95% CI 0.0195–0.0531). Raw paired-end ATAC-seq footprinting provided an orthogonal signal: AP-1 motif intervals scored above same-length motif-free controls selected within the same accessible peaks in every sample, and the AP-1-specific footprint score increased by 0.00509 per dose doubling after replicate adjustment (HC3 95% CI 0.00081–0.00937; exact stratified permutation P = 0.00211). In a matched-background genome-wide scan, related AP-1/bZIP models occupied the nine highest ranks. Within gained chromatin, motif-positive peaks showed greater adjusted overlap with pooled public AP-1 summits (OR 3.82, 95% CI 3.52–4.15); after collapsing related files by study, 71 study clusters gave a random-effects OR of 2.68 (2.51–2.87), with substantial heterogeneity and a prediction interval of 1.58–4.54. Multi-model single-cell and independent bulk-RNA analyses indicated context-dependent downstream AP-1 output, whereas the two valid comparable drug-free CRISPR contrasts were not FDR-significant. Exploratory nearest-TSS mapping of AP-1-rich gained peaks identified 899 protein-coding genes; recurring GO, Reactome and KEGG themes and STRING associations were used only to provide qualitative, hypothesis-generating functional context. Together, the data support a robust association between AP-1-compatible chromatin architecture and olaparib adaptation, while not establishing AP-1 occupancy, necessity or sufficiency in the studied cells.

## 1 Introduction

Cancer cells can survive therapy through non-genetic state transitions that remodel transcriptional programmes and chromatin accessibility before, or in parallel with, stable genetic resistance. Drug-tolerant and persister states have been documented across diverse cancers and therapies, and they frequently involve stress-responsive enhancer reconfiguration, phenotypic plasticity and altered metabolic dependencies (Sharma et al., 2010; Shaffer et al., 2017; Marine et al., 2020; Oren et al., 2021; Russo et al., 2024). The longitudinal Kuramochi ovarian-cancer model developed by França et al. (2024) provides an unusually detailed resistance continuum, with matched chromatin, transcriptomic and functional measurements across escalating olaparib doses. A subsequent conceptual framework proposed that AP-1-family factors may contribute to adaptive regulatory states and chromatin memory during resistance (França and Yanai, 2026).

AP-1 is a family of basic-leucine-zipper dimers assembled from JUN, FOS, ATF and related subunits. AP-1 complexes participate in stress signalling, enhancer selection and signal-dependent chromatin remodelling (Shaulian and Karin, 2002; Vierbuchen et al., 2017), and AP-1-like motifs are enriched in adaptive enhancers in several cancer systems (Zanconato et al., 2015; Zawistowski et al., 2017; Li et al., 2023). Motif enrichment alone, however, is indirect. Local GC content, peak length and baseline accessibility can alter motif detection or public ChIP-seq overlap independently of factor biology. Closely related AP-1/bZIP matrices also recognise many of the same short sequences, so summing model-specific hits can count one underlying motif occurrence repeatedly.

A second inferential gap separates motif architecture from factor engagement and downstream output. Digital genomic footprinting estimates local protection patterns after correction for Tn5 sequence bias, but it remains an indirect occupancy proxy and requires appropriate local controls (Bentsen et al., 2020). Conversely, transcription-factor regulon scores and genetic dependencies report downstream or functional layers that need not be synchronous with motif accessibility. A strong analysis should therefore ask whether AP-1-associated evidence converges across sequence, accessibility, footprint, public-cistrome, transcriptomic and fitness layers, while preserving the evidentiary limits of each.

Here we provide a calibrated and covariate-aware test of AP-1-associated chromatin remodelling along the olaparib-adaptation continuum. We use HOMER motif models, coordinate-level de-duplication, flexible all-peak regression, overlap weighting and matched common-support analysis. We then follow fixed motif-positive/motif-negative peak pairs across dose, reprocess raw paired-end ATAC-seq for within-peak footprinting, compare AP-1 with the complete HOMER known-motif library, and evaluate public AP-1 ChIP overlap after covariate matching and study-level clustering. Multi-model single-cell RNA-seq, independent bulk-RNA datasets and a metabolic CRISPR screen define how transcriptional output and dependency relate to the chromatin signal. Finally, an explicitly exploratory layer maps AP-1-rich gained peaks to nearby GENCODE genes and examines pathway and functional-interaction context. The study remains computational and associational: it identifies AP-1-compatible regulatory architecture but does not directly measure AP-1 occupancy, validate enhancer-target links or establish causal necessity or sufficiency. This layered design is consistent with frameworks that treat therapy adaptation as a dynamic, multilayer state-transition process rather than as a static biomarker problem (França et al., 2024; Dutta and Goli, 2026).

## 2 Results

### 2.1 Coordinate de-duplication removes an artificial high-density AP-1 tail

The original workflow treated hits from eight AP-1/bZIP motif models as separate observations even when their genomic intervals overlapped. Because the matrices recognise closely related sequences, a single element could contribute multiple model-specific hits. Among T320-gained peaks, the summed count averaged 7.11 hits per peak and 9,434 peaks appeared to contain at least 10 hits. After clustering overlapping genomic intervals, the mean fell to 0.81 motif occurrences per gained peak and only one gained peak contained at least 10 occurrences. The previously defined high-density candidate population was therefore a motif-model redundancy artefact and was removed from every downstream analysis.

**FIGURE 1:**
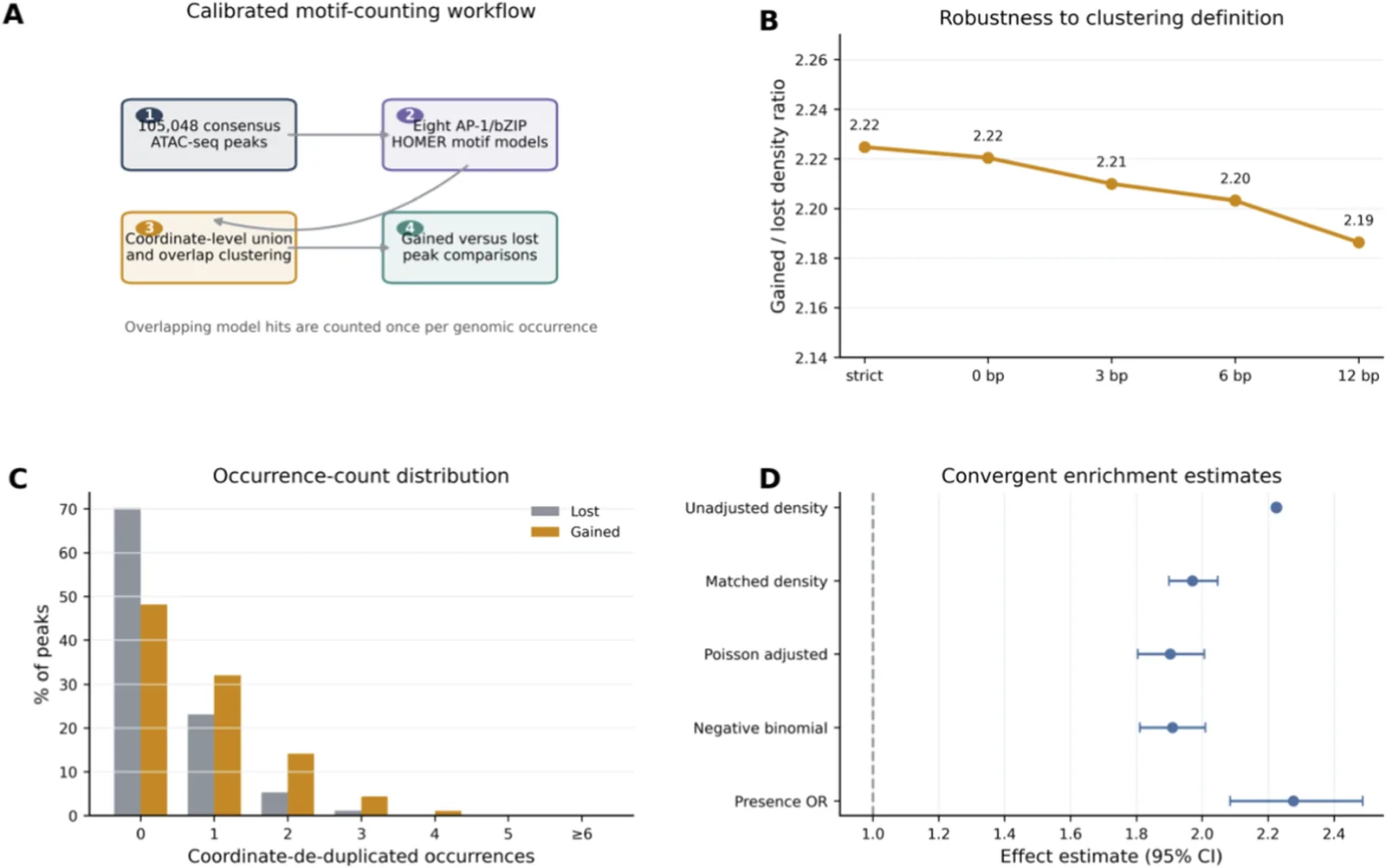
Coordinate-calibrated AP-1 motif architecture. **(A)** Analysis workflow from 105,048 consensus ATAC-seq peaks through eight AP-1/bZIP motif models, coordinate-level union and gained-versus-lost comparison. **(B)** Gained/lost motif-density ratios under strict-overlap and 0–12-bp clustering definitions. **(C)** Distribution of coordinate-de-duplicated AP-1 occurrences in gained and lost peaks. **(D)** Unadjusted, matched and model-adjusted enrichment estimates with 95% confidence intervals. Motif occurrences are sequence matches, not verified occupancy.

The primary definition counted the union of motif intervals under strict genomic overlap. Gained peaks had a mean density of 0.724 AP-1 motif occurrences per kilobase, compared with 0.326 in lost peaks, giving an unadjusted ratio of 2.225. At least one occurrence was present in 51.86% of gained and 29.83% of lost peaks. The estimate was insensitive to clustering gaps of 0, 3, 6 or 12 bp, which gave raw density ratios of 2.220, 2.210, 2.203 and 2.186, respectively (Figure 1; Supplementary Table S1). Throughout, ‘motif occurrence’ denotes a coordinate-clustered sequence match and not an experimentally occupied AP-1 site.

### 2.2 AP-1 motif enrichment survives all-peak adjustment and common-support matching

We next asked whether the approximately twofold raw enrichment could be explained by measured peak-level covariates. The primary all-peak Poisson model used motif-occurrence count as the outcome, included a log peak-length offset, adjusted nonlinearly for GC content, log peak length and baseline accessibility, and used chromosome-clustered covariance. Across all 57,204 classified peaks, the adjusted density rate ratio was 1.90 (95% CI 1.80–2.01). A negative-binomial model that allowed extra-Poisson variation gave 1.88 (1.78–1.98), and the corresponding all-peak logistic model gave an adjusted odds ratio of 2.28 (2.08–2.49) for binary motif presence.

As a design-based complement, overlap weighting reduced the standardised mean differences for GC content, log peak length and baseline accessibility from −0.289, 0.044 and −0.990 to −0.001, −0.001 and −0.004. The overlap-weighted density ratio was 1.95 (95% chromosome-block bootstrap CI 1.82–2.11), with effective weighted sample sizes of 8,869 gained and 8,857 lost peaks.

**FIGURE 2:**
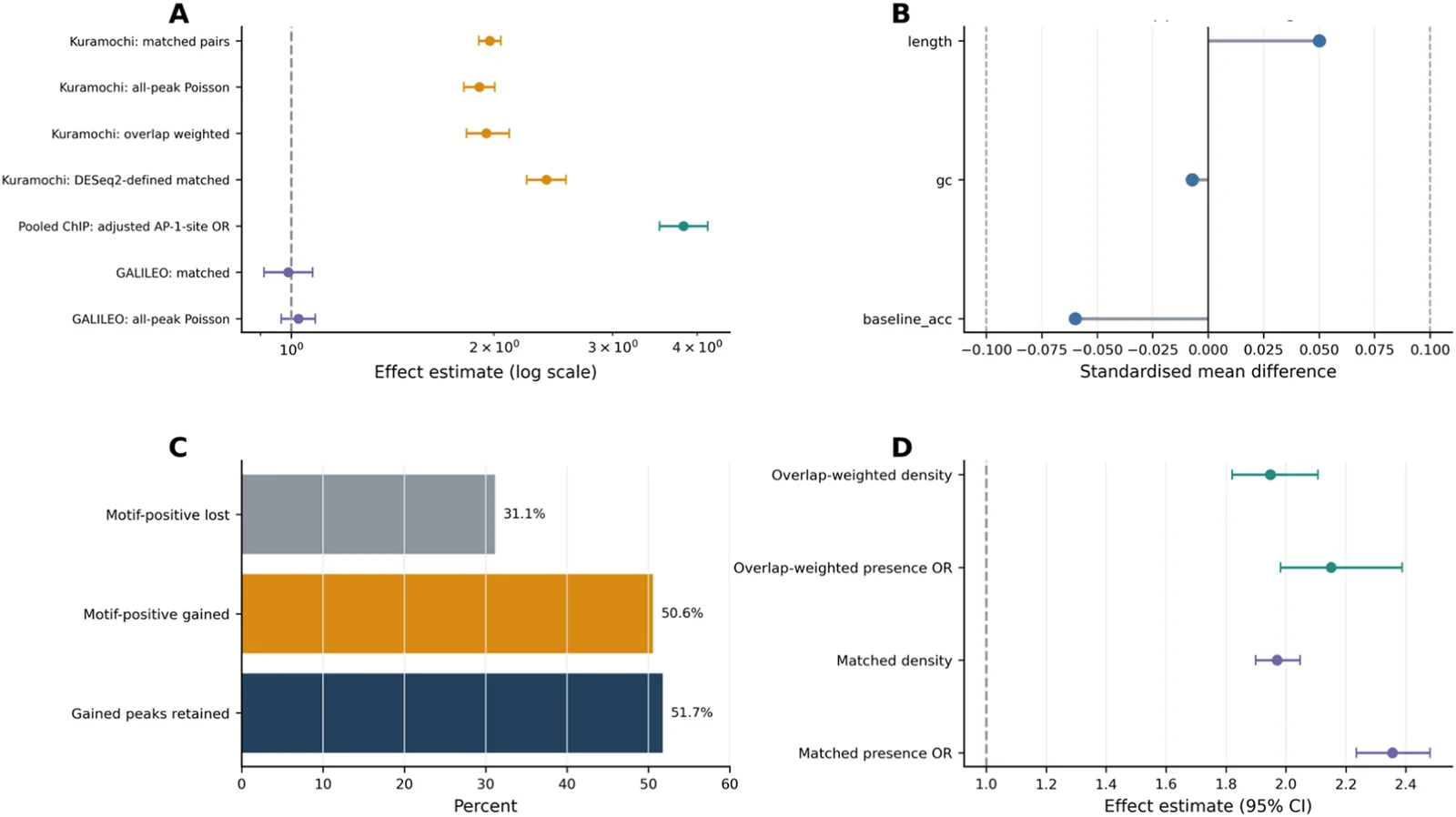
Covariate adjustment and common-support validation. **(A)** Convergence of matched, all-peak, overlap-weighted, replicate-aware, public-ChIP and cross-system sensitivity estimates. The GALILEO paclitaxel estimates are close to unity and are included as a cross-system comparator in which comparable AP-1 motif enrichment was not observed. **(B)** Covariate balance after common-support matching. **(C)** Matching coverage and motif-presence proportions in gained and lost peaks. **(D)** Overlap-weighted and matched density and presence estimates with 95% confidence intervals. The panels distinguish population-average modelling from design-based common-support estimands.

We also matched gained and lost peaks without replacement on GC content, peak length and baseline accessibility. Of 27,154 gained peaks, 14,046 (51.7%) found acceptable lost partners. AP-1 motif density was 0.660 per kilobase in gained peaks and 0.335 in matched lost partners, yielding a paired ratio of 1.97 (95% pair-bootstrap CI 1.90–2.05). Binary occurrence was observed in 50.56% and 31.15% of gained and lost partners, respectively, with a matched odds ratio of 2.36 (2.24–2.48). Because unmatched gained peaks differed materially from the common-support subset, the all-peak adjusted model remains primary. A replicate-aware DESeq2 endpoint sensitivity retained only 3,784 matched pairs and residual imbalance remained; its density ratio of 2.39 (2.24–2.56) is therefore supplementary. Covariate balance for the matched design is reported in Supplementary Table S2. The convergence of the primary regression, negative-binomial, weighting and matching estimates around 1.9–2.0 is the central result (Figure 2; Table 1). As a cross-system comparator, the same coordinate-de-duplicated workflow was applied to the GALILEO breast-cancer paclitaxel dataset. Neither the matched analysis nor the all-peak Poisson model showed comparable AP-1 motif enrichment: the matched density ratio was 0.99 (95% CI 0.91–1.08), and the adjusted all-peak rate ratio was 1.02 (95% CI 0.97–1.09). Thus, the approximately twofold Kuramochi signal was not reproduced in this paclitaxel system.

**TABLE 1:** AP-1 motif-enrichment estimates under complementary definitions and adjustment strategies. Effect types are kept distinct: rate or density ratios apply to motif occurrence per length, whereas odds ratios are used only for binary presence or overlap.

Analysis	Peaks/pairs	Adjustment	Estimate (95% CI)	Effect type
Unadjusted strict-overlap	27,154 gained/30,050 lost	None	2.22 (2.14–2.33)	Density ratio
All-peak Poisson (primary)	57,204 peaks	Splines: GC, log length, baseline accessibility	1.90 (1.80–2.01)	Rate ratio
All-peak negative binomial	57,204 peaks	Same as primary	1.88 (1.78–1.98)	Rate ratio
Overlap weighting	57,204 peaks	GC, log length, baseline accessibility	1.95 (1.82–2.11)	Density ratio
Matched common support	14,046 pairs	GC, length, baseline accessibility	1.97 (1.90–2.05)	Paired density ratio
DESeq2-defined matched sensitivity	3,784 pairs	GC, length, baseline accessibility; residual imbalance	2.39 (2.24–2.56)	Paired density ratio

### 2.3 Fixed AP-1 motif-positive and motif-negative pairs diverge across the adapted dose series

Dose-specific reclassification can create a misleading trajectory because the analysed peak population changes at every dose. We therefore selected AP-1 motif-positive and motif-negative peaks once, matched them exactly by chromosome and within calipers for GC content, peak length and baseline accessibility, and followed the same 35,325 pairs across all deposited ATAC-seq samples. Match balance was strong, with absolute standardised mean differences no greater than 0.025. For each sample, the fixed-pair AP-1 accessibility score was the mean log2-normalised accessibility of motif-positive peaks minus that of matched motif-negative partners.

**FIGURE 3:**
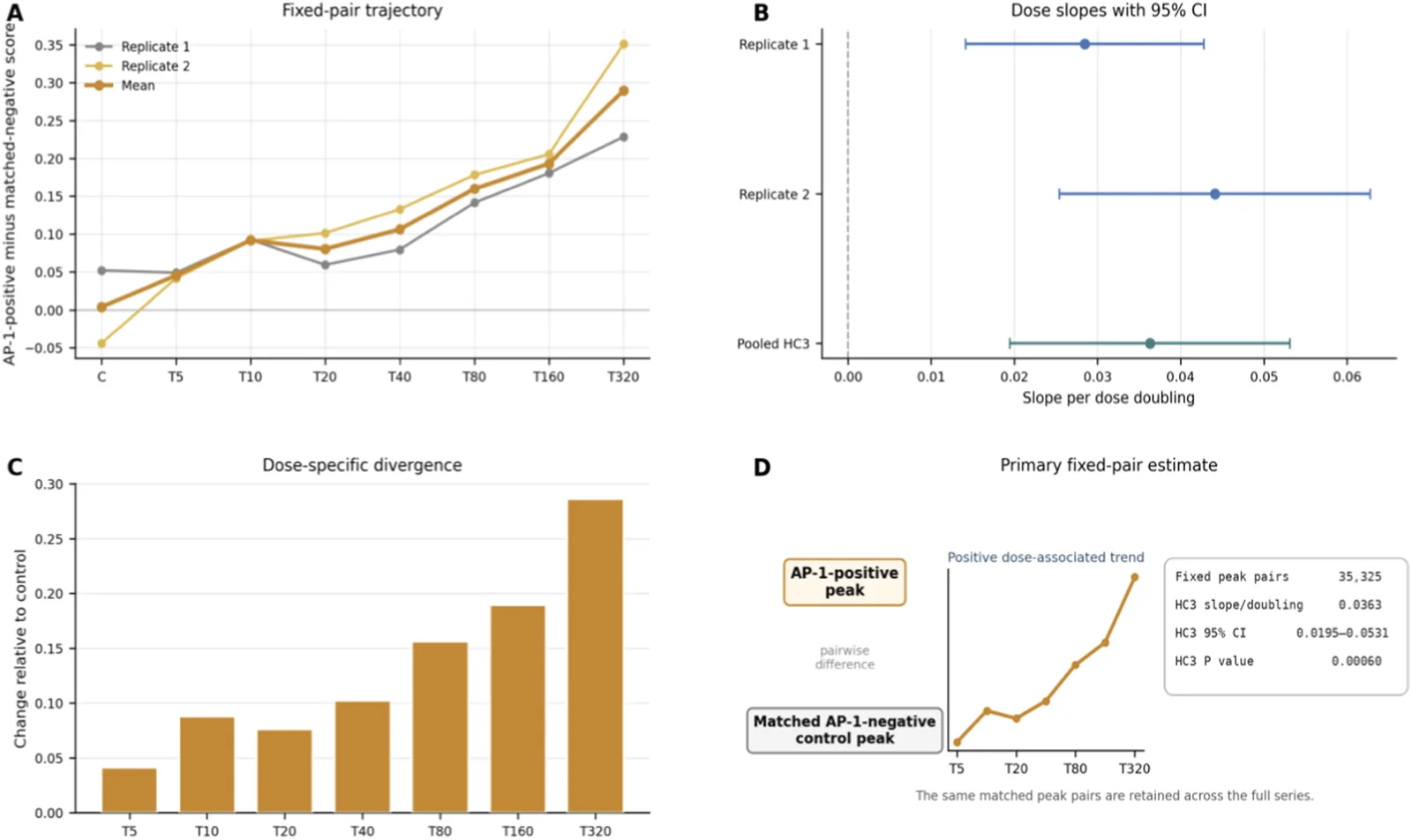
Fixed-pair AP-1-associated accessibility across olaparib adaptation. **(A)** Replicate-resolved and mean trajectories for 35,325 fixed AP-1-positive/matched-negative peak pairs. **(B)** Replicate-specific slopes and the pooled HC3 estimate per dose doubling, with 95% confidence intervals. **(C)** Dose-specific divergence from control. **(D)** Primary HC3 model summary. P = 0.00060 is the small-sample t-reference P value, not a permutation P value. T5-T320 denote adaptation to 5–320 µM olaparib. The overall trend is positive, although intermediate-dose behaviour is not strictly monotonic.

Across the seven adapted doses, the contrast generally increased but was not strictly monotonic at every point. A model of the 14 replicate-level adapted-dose scores with log2 dose and replicate as predictors estimated a gain of 0.0363 contrast units per dose doubling (HC3 95% CI 0.0195–0.0531; HC3 P = 0.00060). Separate replicate slopes were 0.0285 and 0.0441, both positive. Thus, AP-1 motif status is associated with a stronger accessibility trajectory across adaptation, but ‘positive dose-associated trend’ is more accurate than ‘monotonic increase’ (Figure 3; Supplementary Table S3).

### 2.4 Within-peak ATAC footprinting provides orthogonal AP-1-associated evidence

To test whether the motif signal was accompanied by altered local cleavage architecture, we reprocessed raw paired-end ATAC-seq reads for the 16 control and adapted-dose libraries and applied TOBIAS bias correction and footprint scoring. Strict-overlap AP-1 motif clusters were paired with same-length motif-free control intervals drawn from the same accessible peak. The controls did not overlap any AP-1 motif interval, and 56,598 explicit motif-control pairs were defined before score extraction. Depending on library coverage, 54,862–55,599 valid pairs contributed to each sample-level estimate.

AP-1 motif intervals had higher mean footprint scores than their within-peak controls in every sample. The mean AP-1-specific difference was 0.0440 in control, 0.0575 at T5, 0.0609 at T10, 0.0557 at T20, 0.0521 at T40, 0.0624 at T80, 0.0763 at T160 and 0.0925 at T320. Thus, the trajectory showed intermediate-dose attenuation followed by stronger high-dose differences rather than strict monotonicity. Condition-level values are reported in Supplementary Table S4.

**FIGURE 4:**
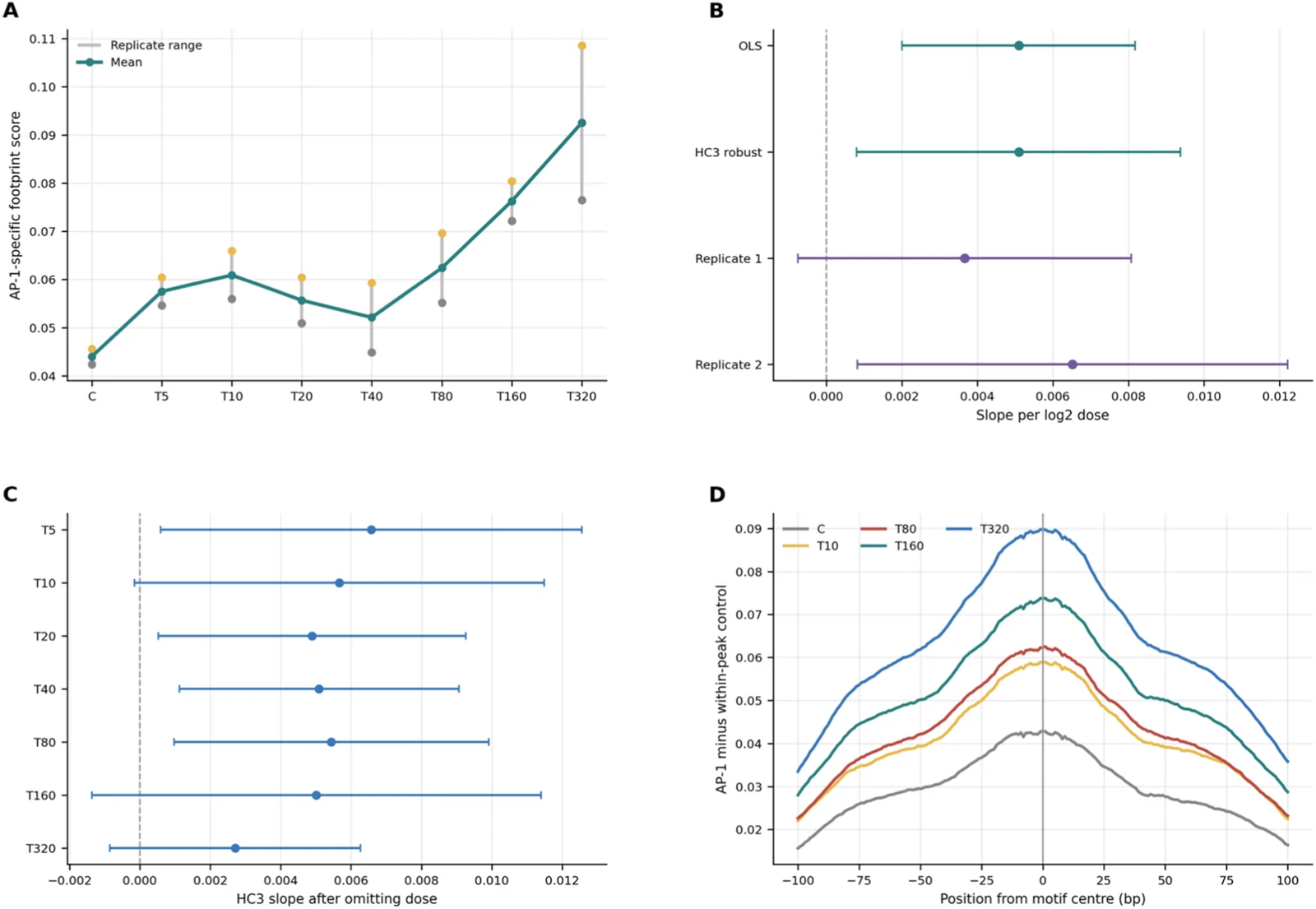
Orthogonal within-peak digital footprinting. **(A)** Mean AP-1-specific footprint trajectory with the two replicate values shown as the replicate range at each condition. **(B)** Conventional, HC3-robust and replicate-specific dose slopes. **(C)** Leave-one-dose-out sensitivity displayed in canonical dose order. **(D)** Motif-centred AP-1-minus-within-peak-control metaprofiles for representative states. T5-T320 denote adaptation to 5–320 µM olaparib. The exact stratified permutation test enumerated all 25,401,600 within-replicate dose assignments.

Using biological samples as the inferential unit, a replicate-adjusted model estimated an increase of 0.00509 AP-1-specific score units per dose doubling. The conventional small-sample 95% CI was 0.00200–0.00818 (P = 0.00398); the HC3 robust interval was 0.00081–0.00937 (P = 0.02398). An exact two-sided permutation test that independently permuted the seven dose labels within each replicate over all 25,401,600 assignments gave P = 0.00211. Both replicate-specific slopes were positive, and every leave-one-dose-out slope remained positive, although uncertainty increased when T160 or T320 was omitted. These results support an overall dose-associated footprint trend while showing that the high-dose states contribute materially to its magnitude (Figure 4; Supplementary Figure S2; Supplementary Table S5).

### 2.5 Closely related AP-1/bZIP motifs dominate the matched genome-wide scan

To assess specificity beyond a curated panel, we ran HOMER’s complete vertebrate known-motif library on matched gained peaks using their matched lost partners as the explicit background. After repeat masking, 12,713 target and 11,470 background sequences entered the analysis. Closely related AP-1/bZIP matrices occupied the first nine ranks: Jun-AP1, Fosl2, Fra2, Fra1, JunB, Fos, Atf3, BATF and a composite AP-1 model. Jun-AP1 occurred in 28.27% of target and 10.64% of background sequences, while the composite AP-1 model occurred in 49.24% and 30.35%, respectively.

**FIGURE 5:**
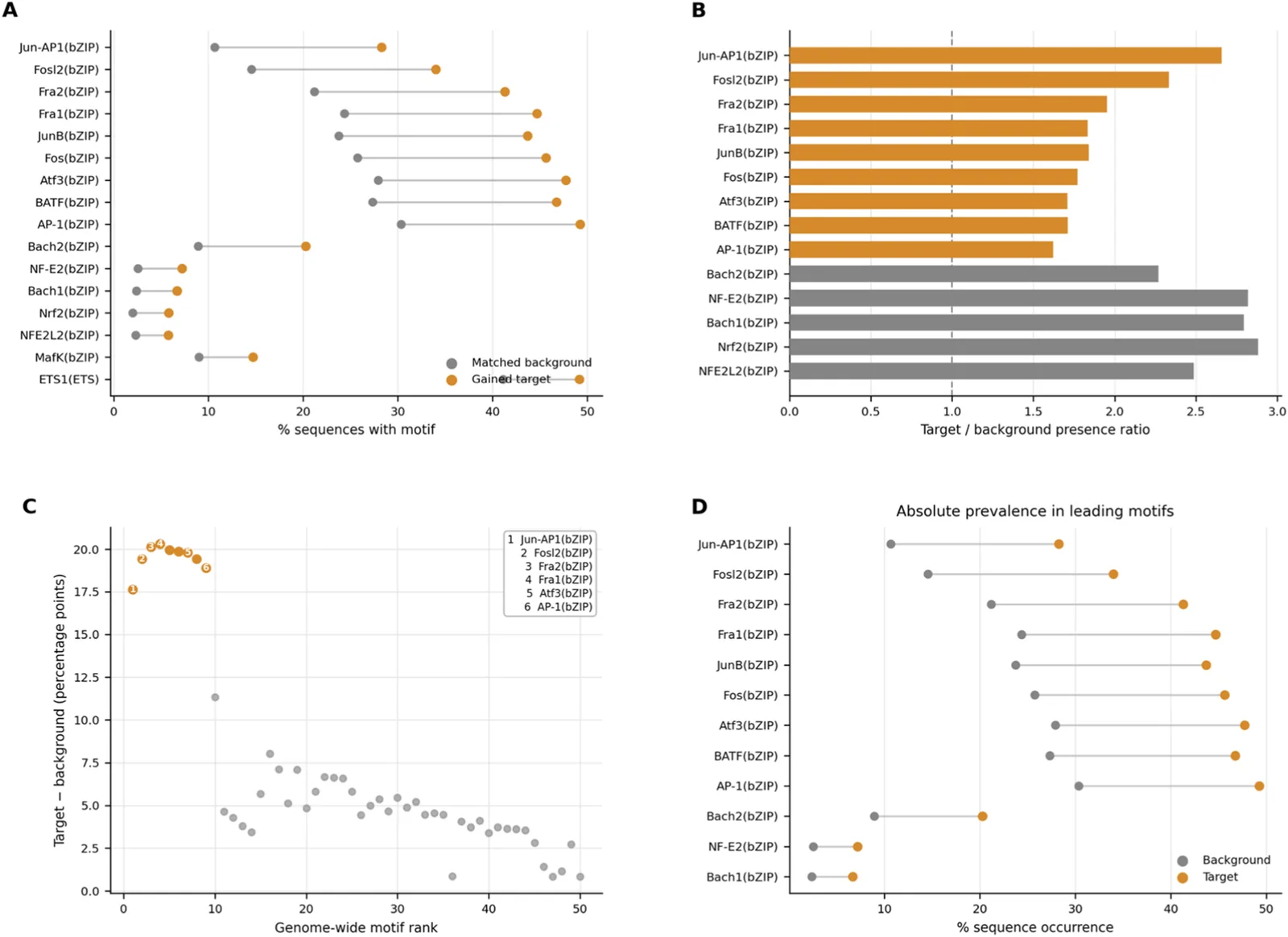
Complete matched-background motif scan. **(A)** Target and matched-background prevalence for the leading 16 HOMER known motifs. **(B)** Target/background presence ratios for the leading 14 motifs, with the first nine AP-1-like/bZIP matrices highlighted. **(C)** Enrichment landscape across the first 50 ranked models; numbered labels identify representative leading AP-1/bZIP matrices without obscuring the data. **(D)** Absolute target and background prevalence for the leading 12 models. Related AP-1 matrices are interpreted collectively rather than as independent subunit evidence.

The top matrices are highly correlated sequence models and are not independent biological confirmations of individual AP-1 subunits. The appropriate interpretation is dominance of AP-1-like/bZIP sequence architecture. Further bZIP motifs occupied ranks 10–15, whereas the leading GATA3-labelled model ranked 259th, occurred in 2.74% *versus* 2.57% of target and background sequences, and was not FDR-significant (q = 0.204; Figure 5 and Table 2).

**TABLE 2:** Top 15 motifs in the matched-background HOMER scan. Related AP-1/bZIP matrices recognise overlapping sequences and are interpreted collectively rather than as independent subunit evidence.

Rank	Motif model	Target %	Background %	Fold presence	BH q
1	Jun-AP1	28.27	10.64	2.66	<10^-300^
2	Fosl2	33.98	14.56	2.33	<10^-300^
3	Fra2	41.30	21.16	1.95	<10^-300^
4	Fra1	44.67	24.36	1.83	<10^-300^
5	JunB	43.70	23.74	1.84	<10^-300^
6	Fos	45.62	25.75	1.77	<10^-300^
7	Atf3	47.72	27.92	1.71	<10^-300^
8	BATF	46.74	27.32	1.71	<10^-300^
9	AP-1	49.24	30.35	1.62	<10^-300^
10	Bach2	20.25	8.92	2.27	<10^-300^
11	NF-E2	7.19	2.55	2.82	<10^-300^
12	Bach1	6.68	2.39	2.79	<10^-300^
13	Nrf2	5.82	2.02	2.88	<10^-300^
14	NFE2L2	5.77	2.32	2.49	<10^-300^
15	MafK	14.69	9.00	1.63	<10^-300^

### 2.6 Public AP-1 cistromic overlap persists after within-gained adjustment and study clustering

Public ChIP-seq cannot measure AP-1 occupancy in olaparib-adapted Kuramochi cells, but it can test whether motif-positive gained sequences tend to be AP-1-bindable in other contexts. Within the gained compartment, AP-1 motif-positive and motif-negative peaks were matched exactly by chromosome and within calipers for GC content, length, baseline accessibility, T320 accessibility and T320 log2 fold change. The 6,405 resulting pairs had absolute standardised mean differences no greater than 0.023 across all five covariates.

Overlap with the pooled public AP-1 summit union occurred in 92.93% of motif-positive peaks and 78.88% of matched motif-negative peaks. The paired odds ratio was 3.83 (95% CI 3.38–4.33), and a spline-adjusted logistic model fitted across all gained peaks produced an almost identical odds ratio of 3.82 (3.52–4.15).

**FIGURE 6:**
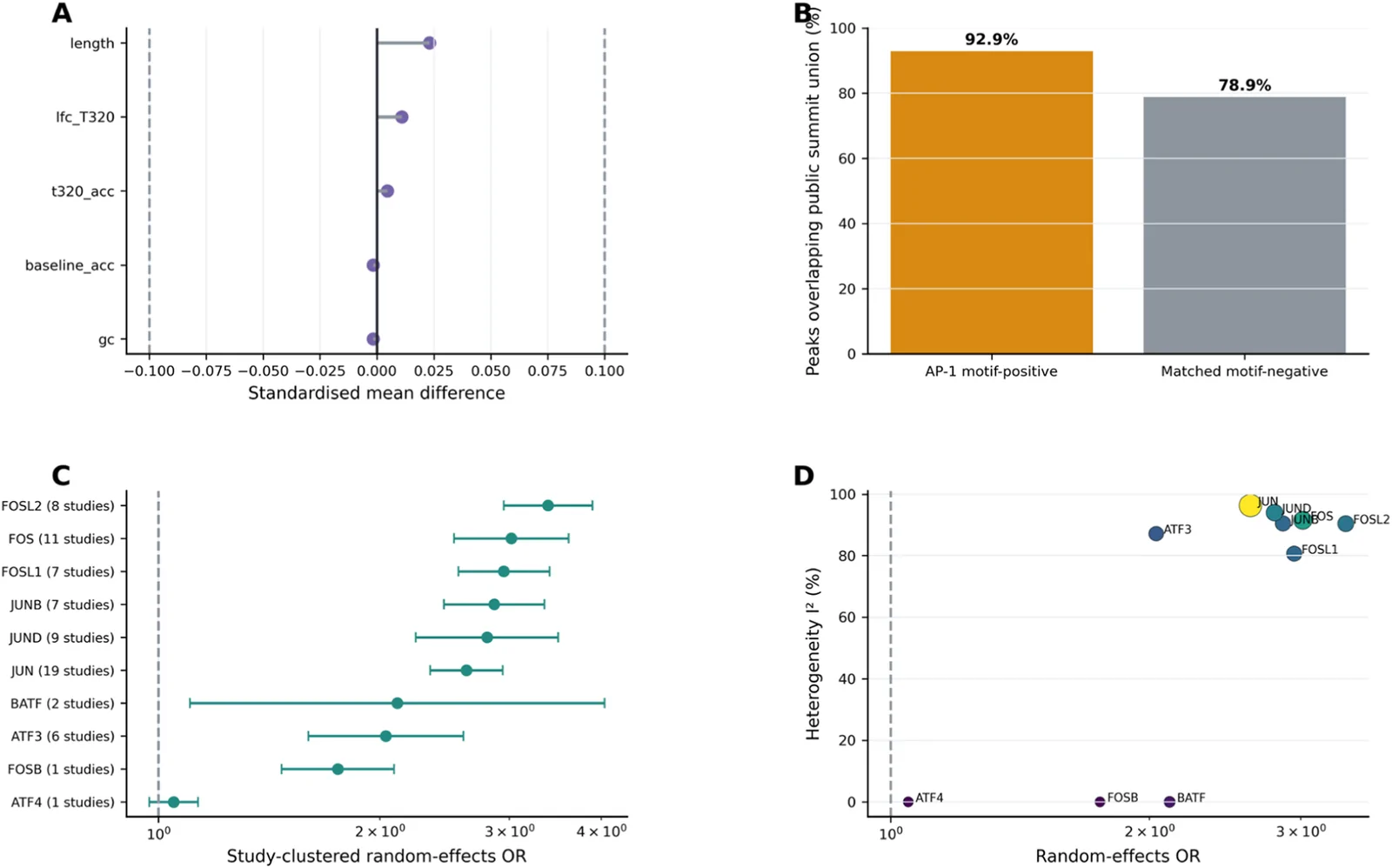
Public AP-1 cistromic support and heterogeneity. **(A)** Balance for 6,405 within-gained motif-positive/motif-negative pairs. **(B)** Pooled public-summit overlap proportions and adjusted odds ratio. **(C)** Factor-labelled random-effects estimates after collapsing related public files by study identifier. **(D)** Relationship between factor-level effect size, study count and I^2^ heterogeneity. Public data quantify cross-context bindability and do not measure occupancy in olaparib-adapted Kuramochi cells.

Because many public files were related replicates or conditions, dataset-level estimates were assigned to study identifiers and collapsed before random-effects pooling. Seventy-one study clusters representing 649 usable peak datasets yielded an overall odds ratio of 2.68 (95% CI 2.51–2.87). Heterogeneity was substantial (I^2^ = 96.7%), and the prediction interval of 1.58–4.54 was much wider than the confidence interval. Factor-labelled study-clustered estimates were strongest for FOSL2 (OR 3.38), FOS (3.02), FOSL1 (2.95), JUNB (2.86), JUND (2.80) and JUN (2.62), while ATF4 was near unity. The consistent positive average association is therefore accompanied by large cross-study variability (Figure 6; Table 3; Supplementary Figure S5).

**TABLE 3:** Study-clustered random-effects summaries of matched public AP-1 peak-dataset effects. The prediction interval describes expected heterogeneity across new study contexts; estimates remain descriptive because study definitions and public processing pipelines differ.

Factor	Studies	Datasets	Random-effects OR	95% CI	Prediction interval	I^2^ (%)
ALL AP-1	71	649	2.68	2.51–2.87	1.58–4.54	96.7
FOSL2	8	97	3.38	2.95–3.89	2.34–4.89	90.4
FOS	11	71	3.02	2.52–3.61	1.74–5.24	91.4
FOSL1	7	29	2.95	2.55–3.40	2.08–4.17	80.7
JUNB	7	79	2.86	2.44–3.35	1.90–4.31	90.4
JUND	9	87	2.80	2.24–3.49	1.51–5.16	94.0
JUN	19	208	2.62	2.34–2.94	1.62–4.25	96.3
ATF3	6	57	2.04	1.60–2.60	1.17–3.55	87.1
ATF4	1	15	1.05	0.97–1.13	0.97–1.13	0.0

These analyses quantify cross-context AP-1 bindability. They do not establish which AP-1 dimer, if any, occupies a locus in olaparib-adapted cells, and they do not convert the motif or footprint results into direct occupancy evidence.

### 2.7 Downstream AP-1 transcriptional output is context dependent; valid CRISPR contrasts are not significant

We next asked whether increasing AP-1-associated chromatin architecture implied continuously increasing downstream AP-1-family output. Condition-level pseudobulk analysis of matched Kuramochi single-cell RNA-seq remained non-monotonic: the AP-1-family composite peaked at T10, stayed positive at T20 and fell below its cross-condition mean at T80-T320. The pattern was not unique to Kuramochi. OVSAHO and COV362 showed early/intermediate increases with later attenuation, while two patient-derived xenograft series showed state-specific reversals rather than a common monotonic trajectory. Because each state contributed one condition-level pseudobulk, these analyses are descriptive. The complete factor-level display is provided in Supplementary Figure S4.

Independent bulk-RNA analysis further indicated that established PARP-inhibitor resistance need not retain high AP-1 transcriptional output. In GSE153867, the AP-1 regulon composite was lower in eight A2780 olaparib-resistant samples than in eight parental samples (mean difference resistant minus parental −0.349; exact permutation P = 0.0137; descriptive Hedges g = −1.33). Two UWB1.289 comparisons in GSE235980 showed concordant decreases in BRCA1-deficient and BRCA1-complemented backgrounds, but each contained only two samples per group and was treated as exploratory.

**FIGURE 7:**
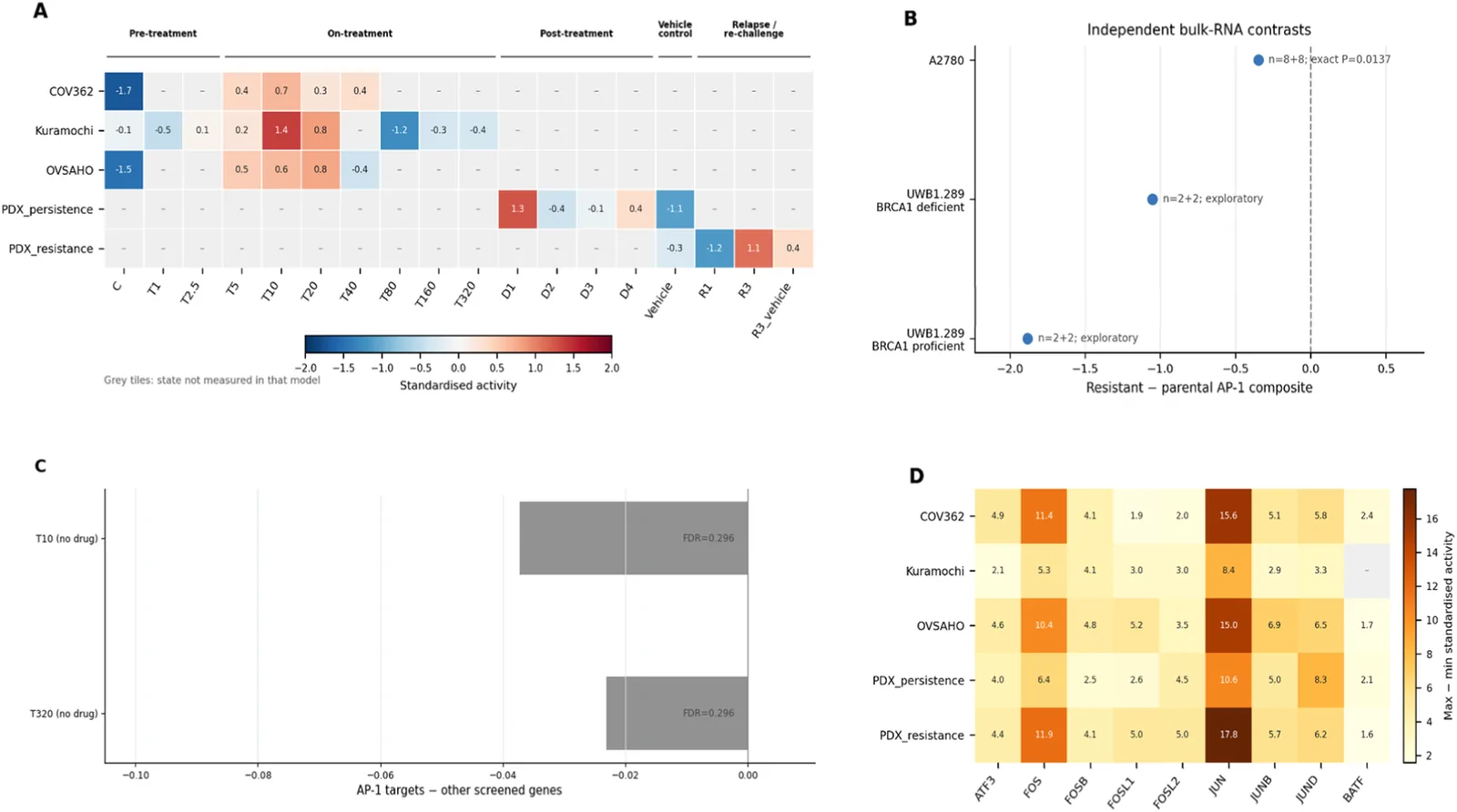
Stage-dependent transcriptional and fitness outputs. **(A)** Condition-level AP-1-family composite activity across Kuramochi, OVSAHO, COV362 and two PDX series; unmeasured model-state combinations are explicitly marked. **(B)** Independent bulk-RNA resistant-versus-parental point estimates; exact permutation inference is shown for A2780, whereas the n = 2 UWB1.289 contrasts are explicitly exploratory. **(C)** AP-1-regulon target-minus-other-gene dependency shifts for the two valid comparable drug-free CRISPR contrasts. T10 and T320 denote cells adapted to 10 and 320 µM olaparib, respectively; no T40 CRISPR state was deposited, and both displayed contrasts had FDR = 0.296. **(D)** Within-model dynamic range (maximum minus minimum standardised activity across measured states) for individual AP-1-family subunits. These orthogonal layers support context dependence and possible temporal rewiring rather than sustained linear activation.

Finally, we re-examined the public metabolism-focused CRISPR screen from the Kuramochi resistance continuum. GEO metadata and the deposited count matrix contain control, T10 and T320 states; no T40 CRISPR state was deposited. We therefore restricted inference to the two comparable drug-free endpoint contrasts, T10 *versus* control and T320 *versus* control. Among 179 measured AP-1-regulon targets, target-minus-other-gene depletion differences were −0.0372 and −0.0230, respectively; neither was significant after Benjamini-Hochberg correction across the two valid contrasts (FDR = 0.296 for both). The drug-treated endpoints were not contrasted because control and T320 cells were screened at different olaparib concentrations. Thus, the CRISPR screen provides no statistically significant stage-specific AP-1-target dependency signal. Collectively, the transcriptomic and functional data support context dependence and possible temporal rewiring rather than sustained linear activation (Figure 7; Table 4).

**TABLE 4:** Orthogonal validation and boundary analyses. These layers have different units and estimands and are not combined into a single meta-analytic effect.

Layer	Comparison	Estimate	Inference	Interpretation
ATAC footprint	14 treated samples across T5-T320	Slope 0.00509 per dose doubling	HC3 CI 0.00081–0.00937; exact P = 0.00211	Overall dose-associated increase with intermediate attenuation
Multi-model scRNA	Kuramochi, OVSAHO, COV362 and two PDX series	Non-monotonic AP-1 composite	Descriptive; one pseudobulk per state	Output is state- and model-dependent
Bulk RNA	A2780 resistant vs. parental, 8 vs. 8	Difference −0.349	Exact permutation P = 0.0137	Established resistance has lower AP-1 composite
Bulk RNA sensitivity	UWB1.289 deficient and proficient, 2 vs. 2	Differences −1.05 and −1.88	Exploratory small-n	Directionally concordant only
Metabolic CRISPR	T10 and T320 vs. C, drug-free	Differences −0.0372 and −0.0230	FDR = 0.296 for both	No significant dependency shift

### 2.8 Exploratory peak-to-gene mapping places AP-1-rich gained chromatin in signalling and stress-response networks

**FIGURE 8:**
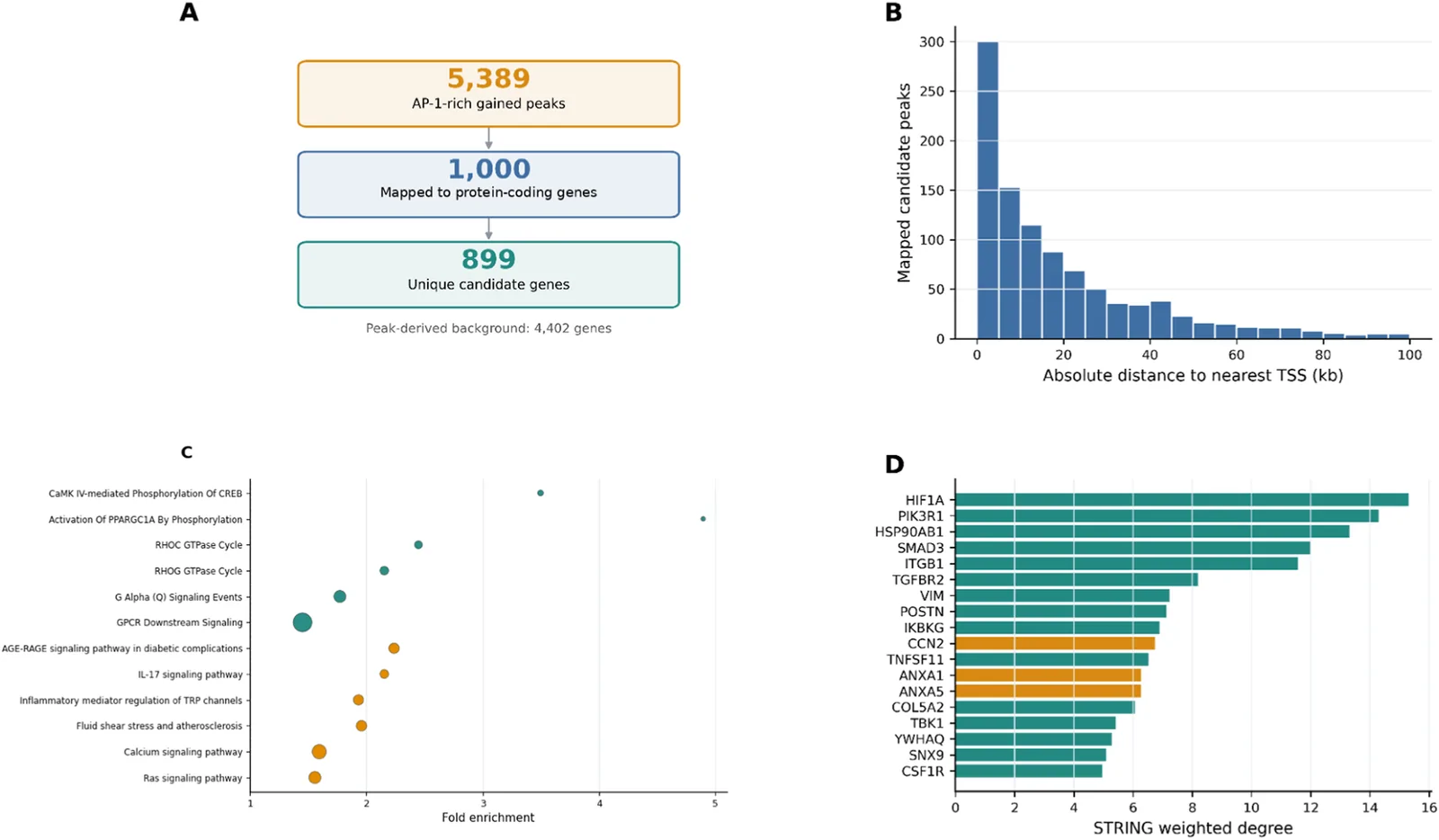
Exploratory functional context and network prioritisation. **(A)** Nearest-GENCODE-TSS mapping flow for AP-1-rich gained peaks. **(B)** Distribution of retained peak-to-TSS distances. **(C)** Representative high-ranking Reactome and KEGG terms from the background-corrected analysis, displayed descriptively by fold enrichment. No pathway-level statistical enrichment or causal pathway activation is inferred; the analysis script, candidate and background gene lists, and pathway-library quality-control summary are supplied in the public code and data release. **(D)** Highest-connectivity STRING candidate-gene hubs. Nearest-TSS assignment does not validate enhancer targets, and STRING associations need not be direct physical interactions.

To provide a restrained functional context without reviving the invalid original high-density class, we defined an exploratory candidate set as gained peaks containing at least two coordinate-de-duplicated AP-1 occurrences. This threshold selected 5,389 peaks. Peak centres were assigned to the nearest GENCODE v49 protein-coding transcription start site within 100 kb. One thousand candidate peaks met these criteria and mapped to 899 unique genes; the corresponding gained-peak background contained 6,266 mapped peaks and 4,402 genes. Repeated nearest-TSS assignments included SACM1L, TRIO and CCN2 (four peaks each), and SEMA3C, DNMBP, MAP3K14, ANXA5, PLOD2, CYP24A1 and TOP1 (three peaks each) (Figure 8A; Supplementary Figure S1; Supplementary Table S6).

Background-corrected ranking across GO Biological Process, Reactome and KEGG yielded recurring descriptive themes involving GPCR/G-protein and PLC signalling, RHO-family GTPase cycles, calcium and TRP-channel regulation, phosphoinositide signalling, oxidative-stress and HIF-associated responses, inflammatory programmes, and extracellular-matrix organisation. These themes are presented only as qualitative, hypothesis-generating functional context; no pathway-level statistical enrichment or causal pathway activation is inferred (Figure 8C; Supplementary Table S7).

A STRING functional-association network contained 199 connected or singleton candidate genes and 325 retained associations. The most connected weighted hubs included HIF1A, PIK3R1, HSP90AB1, SMAD3, ITGB1, TGFBR2, VIM, POSTN, IKBKG and CCN2; prominent retained links included HSP90AB1-HIF1A, SMAD3-TGFBR2, TBK1-IKBKG and MAP3K14-IKBKG (Figure 8D; Supplementary Figure S3; Supplementary Table S8). These analyses are deliberately hypothesis-generating: nearest-TSS assignment does not validate enhancer-gene regulation, the pathway themes are qualitative, and STRING functional associations need not represent direct physical binding (Table 5).

**TABLE 5:** Exploratory functional-context analyses of AP-1-rich gained peaks. These analyses are not part of the primary peak-level test and are not used to infer direct enhancer-target regulation or AP-1 causality.

Layer	Principal result	Quantitative summary	Evidentiary limit
Peak-to-gene mapping	1,000 mapped AP-1-rich peaks; 899 genes	Nearest GENCODE v49 protein-coding TSS within 100 kb	Exploratory assignment, not validated regulation
Exploratory functional context	Recurring pathway themes	GPCR/RHO, calcium/TRP, phosphoinositide, oxidative-stress, HIF, inflammatory and ECM-associated contexts	Qualitative, hypothesis-generating context only; no pathway-level significance or activation inferred
STRING	199 nodes; 325 associations	HIF1A, PIK3R1, HSP90AB1, SMAD3 and ITGB1 among top hubs	Functional association, not necessarily physical interaction

## 3 Discussion

This reanalysis supports a conservative but robust conclusion: chromatin that gains accessibility at the T320 olaparib-adapted endpoint contains approximately twice the coordinate-de-duplicated AP-1 motif-occurrence density of chromatin that loses accessibility, and the association is not explained by measured differences in GC content, peak length or baseline accessibility. The strongest evidence is estimator convergence rather than any single P value: the all-peak Poisson, negative-binomial, overlap-weighted and common-support estimates were 1.90, 1.88, 1.95 and 1.97, respectively.

The analytical correction is central to the paper. Eight related AP-1/bZIP matrices often identify the same short genomic sequence. Summing model-specific hits created an apparently high-density class that disappeared after coordinate-level de-duplication. Primary inference therefore treats motif content as a continuous or binary peak-level property across the full gained/lost compartments. A separate functional-context analysis was retained only after recalibration: gained peaks with at least two non-redundant occurrences were mapped to nearby GENCODE genes explicitly as a hypothesis-generating layer, not as a privileged mechanistic class.

Two independent ATAC-based analyses add a dynamic dimension. Fixed motif-positive/motif-negative peak pairs became more accessible across the adapted dose series, and raw-read footprinting showed increasing AP-1 motif-centred scores relative to same-peak motif-free controls. Neither trajectory was perfectly monotonic. In both cases, intermediate-dose attenuation preceded stronger T160-T320 differences. The exact within-replicate permutation result makes the footprint trend less dependent on small-sample parametric assumptions, but the two-replicate design remains a limitation and the high-dose states materially influence the slope.

The matched genome-wide scan and public-cistrome analyses strengthen sequence-level specificity while also defining its limits. Related AP-1/bZIP matrices dominated the highest ranks against a matched lost-peak background, whereas GATA3 was near null. Motif-positive gained peaks also overlapped public AP-1 summits more frequently after adjustment. Collapsing related files by study reduced pseudoreplication, but heterogeneity remained extreme and the prediction interval was broad. Public peak collections therefore index recurrent cross-context bindability, not AP-1 occupancy in Kuramochi cells.

The exploratory functional layer produced a coherent but non-exclusive hypothesis-generating landscape involving oxidative-stress, GPCR/PLC, phosphoinositide, calcium, RHO-family, HIF-associated and extracellular-matrix contexts. Network links such as MAP3K14-IKBKG, TBK1-IKBKG and SMAD3-TGFBR2 suggest candidate routes for perturbation, but these patterns cannot determine whether AP-1-rich peaks regulate the assigned genes or whether the network is specific to olaparib adaptation. Nearest-TSS assignment can miss distal, multi-target and context-dependent enhancer-gene relationships, so the mappings are used only for prioritisation (Fulco et al., 2019). More broadly, cancer-associated chromatin regulators can exert transcriptional effects outside canonical genomic roles, including non-telomeric TRF2-dependent regulation of IL1R1 and TERT (Mukherjee et al., 2024; Sengupta et al., 2025). These examples illustrate regulatory noncanonicity but do not validate the specific peak-gene assignments made here.

The transcriptomic and CRISPR analyses provide an important boundary. AP-1-family target-expression activity rose in several early or intermediate states but attenuated in later Kuramochi, OVSAHO and some PDX states. Established A2780 and UWB1.289 resistance also showed lower bulk AP-1 composite scores. Neither valid comparable drug-free CRISPR contrast was FDR-significant. Motif enrichment, accessibility, digital footprinting, factor occupancy, target expression and genetic dependency represent distinct molecular layers and need not be synchronous. A plausible temporal model is that AP-1-compatible regulatory architecture becomes accessible during adaptation, while downstream AP-1 output is later attenuated, redistributed among family members or replaced by other programmes. The current data cannot distinguish priming, memory, consequence or causation.

Several limitations remain. The primary chromatin evidence comes from one cell-line/drug system with two biological replicates per ATAC-seq condition. Approximately half of gained peaks lacked a suitable matched lost partner, so the all-peak adjusted analysis is primary. Digital footprint scores are sequence- and cleavage-model-based proxies rather than direct protein measurements. Public ChIP datasets are heterogeneous, and study clustering cannot eliminate all dependence or context mismatch. Multi-model scRNA states have one pseudobulk each, the two UWB1.289 bulk contrasts are very small, and neither valid comparable CRISPR contrast is FDR-significant. No matched AP-1 CUT&RUN/CUT&Tag, family-member perturbation or chromatin-contact data were available. This omission is important because somatic rearrangements can disrupt chromatin-folding domains in cancer (Akdemir et al., 2020), and drug-resistant cells can undergo extensive three-dimensional genome reorganisation with AP-1 enrichment at gained chromatin loops (Wang et al., 2022). The nearest-TSS functional analysis discards long-range and multi-target enhancer relationships, the 100-kb threshold is an operational filter, and the functional themes are descriptive rather than pathway-level evidence. STRING integrates heterogeneous evidence and includes functional associations that may not be direct physical interactions.

The decisive next experiments are family-member-resolved CUT&RUN or CUT&Tag across intermediate and high-dose Kuramochi states; JUN, JUNB, JUND and FOSL1 perturbation followed by ATAC-seq, RNA-seq and drug-response measurement; contact-informed enhancer-gene assignment using Hi-C, Micro-C or activity-by-contact approaches (Fulco et al., 2019); and targeted tests of the HIF1A/PIK3R1, SMAD3/TGFBR2, TBK1/IKBKG and CCN2-associated modules prioritised here. Within present limits, the study provides a reproducible template for cistromic inference: calibrate motif scanning, de-duplicate redundant models by coordinate, diagnose covariate imbalance, combine all-population and common-support estimands, use local controls for footprinting, collapse related public datasets at the study level, and keep sequence bindability, inferred engagement, transcriptional output and causal function conceptually separate.

## 4 Materials and methods

### 4.1 Public datasets and analysis scope

Kuramochi ATAC-seq and matched single-cell RNA-seq data were obtained from GEO accessions GSE247685 and GSE206125, respectively, associated with the resistance-continuum study of França et al. (2024). The ATAC count matrix contained 105,048 consensus peaks across control (C), seven dose-adapted conditions (T5, T10, T20, T40, T80, T160 and T320; labels denote adaptation concentrations of 5, 10, 20, 40, 80, 160 and 320 µM olaparib) and acute exposures, with two replicates per condition. Raw paired-end ATAC sequencing runs were retrieved for C and the seven adapted doses. Additional single-cell models were OVSAHO, COV362 and two PDX series supplied with the public resistance-continuum resources. Independent bulk-RNA datasets were GSE153867 and GSE235980. The metabolism-focused CRISPR screen was GSE247686. The GALILEO breast-cancer paclitaxel dataset was GSE205230. Public AP-1-labelled ChIP-seq peak datasets were obtained from ENCODE and ChIP-Atlas (Oki et al., 2018; ENCODE Project Consortium et al., 2020).

### 4.2 ATAC-seq preprocessing and endpoint groups

Peak coordinates were normalised to chromosome, start and end fields, and a coordinate-derived peak identifier was used throughout. Peak length was end minus start. GC content was calculated from the hg38 reference. Count columns were normalised by median-of-ratios size factors and transformed as stated for each analysis. Baseline accessibility was the log1p-transformed mean raw count of the two controls; T320 accessibility was defined analogously. Peaks with endpoint log2 fold change >0.5 were classified as gained (n = 27,154), and peaks with log2 fold change < -0.5 as lost (n = 30,050).

### 4.3 Calibrated motif scanning and coordinate de-duplication

AP-1 motif occurrences were generated with HOMER v5.1 (Heinz et al., 2010). Eight AP-1/bZIP models (Jun-AP1, Fosl2, Fra1, Fra2, JunB, Fos, BATF and Atf3) were scanned with annotatePeaks.pl using hg38, -size given, -mask and -mbed to emit genomic intervals. Intervals were intersected with ATAC peaks using BEDTools (Quinlan and Hall, 2010), duplicate records were removed, and the primary count was the number of strict-overlap clusters formed from the union of all eight models within each peak. Sensitivity counts allowed gaps of 0, 3, 6 or 12 bp.

### 4.4 All-peak regression and overlap weighting

Analyses were restricted to the 57,204 gained or lost peaks. The primary model was a Poisson generalised linear model of occurrence count with gained status as the exposure, log peak length in kilobases as an offset, and cubic B-spline terms for GC content, log peak length and baseline accessibility. Standard errors were clustered by chromosome. A binomial model with the same adjustment estimated binary motif-presence odds ratios. A discrete negative-binomial model was fitted as a sensitivity. For overlap weighting, a logistic propensity model used spline-transformed GC content, log peak length and baseline accessibility; gained peaks received weight 1-e and lost peaks weight e (Li et al., 2018). Confidence intervals used 2,000 chromosome-block bootstrap resamples.

### 4.5 Matched common-support and DESeq2 sensitivity analyses

Gained peaks were greedily matched without replacement to lost peaks on standardised GC content, peak length and baseline accessibility. Ten random matching orders were evaluated and the solution with the most pairs and best balance was retained. Density-ratio confidence intervals were obtained by resampling complete pairs 5,000 times; binary presence used exact McNemar inference. A replicate-aware PyDESeq2 implementation of the DESeq2 framework (Love et al., 2014) was applied to two controls and two T320 samples using |log2 fold change| > 1 and adjusted P < 0.05; because matching retained 29.9% and residual imbalance remained, this analysis was designated supplementary. For the GALILEO comparator, gained and lost peaks were analysed with the same coordinate-de-duplicated AP-1 definition. The matched analysis balanced GC content and peak length, and the all-peak Poisson model included a log peak-length offset and adjustment for available peak-level covariates.

### 4.6 Fixed-pair accessibility trajectory

All AP-1 motif-positive peaks were matched to motif-negative peaks exactly by chromosome and within calipers of 0.02 GC units, 250 bp length and 0.20 baseline-accessibility units. The same 35,325 pairs were followed across the ATAC series. Counts were median-of-ratios normalised and transformed as log2(normalised count +1). The adapted-dose trend was fitted to the 14 T5-T320 replicate-level scores as score ∼ log2(dose) + replicate, with HC3 standard errors and a small-sample t reference.

### 4.7 Raw-read alignment and digital footprinting

Sixteen paired-end ATAC libraries were aligned to hg38 with Bowtie2 using very-sensitive paired-end settings. Reads were name-sorted, mate information repaired, coordinate-sorted and duplicate-removed with SAMtools. Final BAMs retained MAPQ ≥30 alignments after excluding unmapped, mate-unmapped, secondary, QC-failed and duplicate records. Tn5 bias correction and continuous footprint scoring used TOBIAS v0.17.3 ATACorrect and ScoreBigwig (Bentsen et al., 2020). Strict-overlap AP-1 clusters were paired one-to-one with same-length motif-free intervals within the same accessible peak. Control intervals were required not to overlap any AP-1 motif interval. The sample score was the mean AP-1 interval score minus the mean paired-control score across valid pairs.

### 4.8 Footprint dose inference

The primary footprint trend used the 14 treated biological samples and fitted AP-1-specific score ∼ log2(dose) + replicate. Conventional and HC3 confidence intervals were reported. For an exact two-sided sensitivity, dose labels were permuted independently within each biological replicate and all 7! × 7! = 25,401,600 assignments were enumerated. Separate replicate slopes and leave-one-dose-out fits were also calculated. Region-level bootstrap intervals were used only to describe variability across genomic pairs within a sample and were not treated as biological-replicate uncertainty.

### 4.9 Matched-background genome-wide motif enrichment

Matched gained peaks and paired lost partners were analysed with findMotifsGenome.pl against the complete HOMER vertebrate known-motif library. Matched lost peaks were supplied as the explicit background. Repeat masking left 12,713 target and 11,470 background sequences. Target/background percentages and Benjamini-adjusted q values were parsed for all 472 models, and related bZIP matrices were interpreted at the family level.

### 4.10 Public AP-1 ChIP-seq overlap and study clustering

A pooled union of AP-1-labelled summit intervals from ENCODE and ChIP-Atlas was intersected with authoritative peaks. Within gained chromatin, motif-positive and motif-negative peaks were matched exactly by chromosome and within calipers for GC content, length, baseline accessibility, T320 accessibility and T320 log2 fold change. Pooled overlap used exact McNemar inference; an all-gained binomial model adjusted flexibly for the same covariates with chromosome-clustered covariance. For cross-dataset synthesis, each public BED file was intersected separately with the 6,405 pairs. Related files were assigned to study identifiers and collapsed to one study-level effect before DerSimonian-Laird random-effects pooling (DerSimonian and Laird, 1986). Confidence intervals, prediction intervals, I^2^ and leave-one-study-out diagnostics were calculated overall and by factor label.

### 4.11 Single-cell and bulk-RNA regulon analyses

Expression matrices were aligned to metadata by cell barcode and pseudobulked by condition. CollecTRI regulons (Müller-Dott et al., 2023) were restricted to AP-1-family sources with at least 10 measured targets. Gene expression was standardised across states and combined using signed target weights; TF scores were standardised and averaged to form the AP-1-family composite. Kuramochi, OVSAHO, COV362 and two PDX models were analysed descriptively because each state contributed one pseudobulk. For GSE153867, only the eight A2780-resistant and eight A2780-parental samples were included; C13* shCON and shCEBPB samples were excluded. GSE235980 contrasts were mapped explicitly to BRCA1-deficient/proficient parental and resistant samples. Exact label-permutation inference was used when at least three samples per group were available; n = 2 contrasts were descriptive.

### 4.12 CRISPR integration

GSE247686 guide counts and the human metabolic-library annotation were mapped to gene symbols. The deposited design contains control, T10 and T320 states. Gene-level depletion scores were calculated from end relative to initial guide abundance. Primary inference was restricted to the comparable drug-free T10-versus-control and T320-versus-control contrasts; no T40 screen exists in the deposited design, and drug-treated control and T320 endpoints were not compared because they used different olaparib concentrations. AP-1-regulon targets with measured guides were compared with other screened genes by mean score difference and stratified label permutation. Benjamini-Hochberg correction was applied across the two valid prespecified drug-free contrasts.

### 4.13 Exploratory peak-to-gene, pathway and functional-network analysis

AP-1-rich candidate peaks were defined *post hoc* for exploratory functional context as T320-gained peaks with at least two coordinate-de-duplicated AP-1 occurrences. Peak centres were assigned to the nearest GENCODE v49 gene-level transcription start site after restricting to protein-coding genes. Assignments with absolute distance greater than 100 kb were excluded. Candidate genes were summarised by number of assigned peaks, maximum AP-1 occurrence count, minimum TSS distance, median baseline accessibility and median T320 log2 fold change. The background list comprised protein-coding genes assigned under the same distance rule to all T320-gained peaks. Nearest-TSS assignment was used only for prioritisation and was not interpreted as validated enhancer-target regulation (Frankish et al., 2021).

Functional-context analysis used the GO Biological Process 2023, Reactome 2022 and KEGG 2021 Human gene-set libraries obtained in GMT format from Enrichr (Kuleshov et al., 2016). The 899 candidate genes were evaluated relative to the experimental universe of 4,402 protein-coding genes assigned from all T320-gained peaks under the same GENCODE v49 nearest-TSS and 100-kb distance criteria. Candidate genes and pathway gene sets were intersected with this universe and evaluated using one-sided hypergeometric testing. The public code and data release provides the background-corrected analysis script, the 899-gene candidate list, the 4,402-gene experimental universe and the pathway-library quality-control summary. In the manuscript, recurring themes are presented only qualitatively and no pathway-level significance or activation claim is made. STRING v12 functional associations were retrieved for candidate genes at combined score at least 0.40, and the displayed network emphasised the connected high-weight component and weighted-degree hubs (Szklarczyk et al., 2023). STRING edges were interpreted as functional associations, not necessarily direct physical interactions.

### 4.14 Software and reproducibility

Analyses used Python 3.12.9 or 3.12.13 with NumPy, pandas, SciPy, statsmodels, scikit-learn, PyDESeq2, pyfaidx, pyBigWig, BEDTools, Bowtie2, SAMtools, SRA Toolkit, HOMER v5.1 and TOBIAS v0.17.3, GENCODE v49, Enrichr gene-set libraries and STRING v12. The associated public Zenodo release provides the Python analysis scripts, dataset-accession metadata, the 899-gene candidate and 4,402-gene background lists, and compact machine-readable result tables supporting the principal reported estimates. Raw public datasets, large intermediate matrices, scheduler-specific files, finished figures and aesthetic layout code are not duplicated in the release.

## 5 Data and code availability

Kuramochi ATAC-seq, single-cell RNA-seq and CRISPR-screen data are available through GEO under GSE247685, GSE206125 and GSE247686, respectively. Additional single-cell validation datasets are available under GSE247689 and GSE247690, independent bulk-RNA datasets under GSE153867 and GSE235980, and the GALILEO paclitaxel ATAC-seq comparator under GSE205230. Public AP-1 ChIP-seq peak datasets were obtained from ENCODE and ChIP-Atlas. The associated public code and data release is openly available through Zenodo, record 21520054 (Dutta, 2026; doi:10.5281/zenodo.21520054). It contains Python analysis scripts, dataset-accession metadata, the pathway candidate and background gene lists, and compact machine-readable result tables supporting the principal analyses. Public raw sequencing data are not duplicated and remain available from the source repositories listed above.

## Data Availability

All datasets analysed in this study are publicly available. Kuramochi ATAC-seq, single-cell RNA-seq and CRISPR-screen data are available through GEO under accession numbers GSE247685, GSE206125 and GSE247686, respectively. Additional single-cell validation datasets are available under GSE247689 and GSE247690, independent bulk-RNA datasets under GSE153867 and GSE235980, and the GALILEO paclitaxel ATAC-seq comparator under GSE205230. Public AP-1 ChIP-seq datasets were obtained from ENCODE and ChIP-Atlas. The associated analysis code and supporting machine-readable data are openly available through Zenodo, record 21520054, DOI: 10.5281/zenodo.21520054.
