# Supplementary material for: Covariate-adjusted AP-1 motif architecture and footprinting track olaparib-adaptive chromatin remodelling
Source: Front Syst Biol. 2026 Sep 9;6:1859256. doi: 10.3389/fsysb.2026.1859256 (PMC13597331; doi:10.3389/fsysb.2026.1859256)
Supplement: Supplementary file 1 [file Supplementaryfile1.docx]

Supplementary Material

# Supplementary Methods

## S1. Peak-to-gene assignment

T320-gained peaks containing at least two coordinate-de-duplicated AP-1 occurrences were used only for exploratory functional context. Peak centres were mapped to the nearest gene-level TSS in GENCODE v49 after restricting to protein-coding genes and requiring an absolute TSS distance of 100 kb or less. The same assignment rule was applied to all gained peaks to construct a peak-derived background gene list. Nearest-TSS mapping does not establish enhancer-target regulation and can miss long-range, multi-target and chromatin-contact-dependent relationships.

## S2. Functional enrichment

The 899 candidate genes were evaluated against the 4,402-gene peak-derived universe using GO Biological Process 2023, Reactome 2022 and KEGG 2021 Human gene-set libraries. Candidate and gene-set symbols were intersected with the experimental universe and assessed using one-sided hypergeometric testing. The public code and data release supplies the background-corrected analysis script, candidate and background gene lists, and pathway-library quality-control summary. In the manuscript and supplementary material, the pathway results are presented only as qualitative, hypothesis-generating themes; no pathway-level significance or causal activation is inferred.

## S3. STRING functional network

STRING v12 functional associations were retrieved for mapped candidate genes using a minimum combined score of 0.40. Network summaries include weighted degree, edge count and number of assigned candidate peaks. The main-text display emphasises the connected high-weight hub component; the supplementary display retains the broader retained network, including small components and isolated nodes. STRING associations integrate multiple evidence channels and need not represent direct physical interactions.

## S4. Motif-centred footprint metaprofiles

For each sample, TOBIAS footprint-score BigWigs were averaged in a ±100-bp window around strict-overlap AP-1 motif centres and around matched within-peak motif-free control centres. Condition-level curves are descriptive averages across the two biological replicates. The heatmap displays the AP-1 profile minus the within-peak control profile at each position.

# Supplementary Figures

## Supplementary Figure S1

Supplementary Figure S1. Candidate-gene prioritisation diagnostics. (A) Genes receiving repeated AP-1-rich gained-peak assignments. (B) Relationship between minimum nearest-TSS distance, median T320 accessibility change and maximum AP-1 occurrence count. (C) AP-1 occurrence multiplicity among mapped candidate peaks. (D) Candidate genes with the highest median T320 accessibility change. These panels describe nearest-TSS prioritisation and do not validate enhancer-target regulation.

## Supplementary Figure S2

Supplementary Figure S2. Motif-centred footprint metaprofiles. (A) Mean TOBIAS profiles around AP-1 motifs and same-peak motif-free controls for representative states. Within-peak control profiles are lower and comparatively flat. (B) AP-1-minus-control heatmap across the complete adapted series; the motif-centred difference is strongest at T160-T320.

## Supplementary Figure S3

Supplementary Figure S3. STRING functional-association network of AP-1 candidate genes. Node size reflects weighted degree; orange nodes received multiple candidate-peak assignments. Edges represent retained STRING functional associations at combined score at least 0.40 and need not be direct physical interactions.

## Supplementary Figure S4

Supplementary Figure S4. AP-1-family regulon activities across all analysed single-cell models and states. Rows are model-state pseudobulks and columns are AP-1-family factors. The heatmap emphasises non-monotonic and model-specific activity patterns; these condition-level summaries are descriptive.

## Supplementary Figure S5

Supplementary Figure S5. Public-ChIP heterogeneity diagnostics. (A) Study-clustered factor estimates with confidence and prediction intervals. (B) Number of independent study clusters contributing to each factor label. The wide prediction intervals and high I² values motivate cautious cross-context interpretation.

# Supplementary Tables

Supplementary Table S1. Sensitivity of AP-1 motif quantification to coordinate-clustering definition.

| **Definition** | **Gained density** | **Lost density** | **Gained/lost ratio** | **Gained present** | **Lost present** |
| --- | --- | --- | --- | --- | --- |
| Strict overlap | 0.724 | 0.326 | 2.225 | 51.86% | 29.83% |
| 0 bp gap | 0.722 | 0.325 | 2.220 | 51.86% | 29.83% |
| 3 bp gap | 0.717 | 0.325 | 2.210 | 51.86% | 29.83% |
| 6 bp gap | 0.713 | 0.324 | 2.203 | 51.86% | 29.83% |
| 12 bp gap | 0.706 | 0.323 | 2.186 | 51.86% | 29.83% |

Supplementary Table S2. Covariate balance in the common-support matched gained/lost peak analysis.

| **Feature** | **Gained mean** | **Matched lost mean** | **SMD** |
| --- | --- | --- | --- |
| GC content | 0.4802 | 0.4808 | -0.0073 |
| Peak length (bp) | 1277 | 1250 | 0.0500 |
| Baseline accessibility | 4.625 | 4.672 | -0.0599 |

Supplementary Table S3. Fixed-pair AP-1 accessibility scores across conditions and replicates. P10 and P320 are the deposited acute-exposure labels.

| **Sample** | **Condition** | **Replicate** | **Score** | **Region-level CI low** | **Region-level CI high** |
| --- | --- | --- | --- | --- | --- |
| C_1 | C | 1 | 0.0521 | 0.0488 | 0.0560 |
| T5_1 | T5 | 1 | 0.0489 | 0.0408 | 0.0589 |
| T10_1 | T10 | 1 | 0.0924 | 0.0835 | 0.1012 |
| T20_1 | T20 | 1 | 0.0594 | 0.0503 | 0.0673 |
| T40_1 | T40 | 1 | 0.0794 | 0.0681 | 0.0892 |
| T80_1 | T80 | 1 | 0.1414 | 0.1285 | 0.1539 |
| T160_1 | T160 | 1 | 0.1808 | 0.1654 | 0.1940 |
| T320_1 | T320 | 1 | 0.2284 | 0.2143 | 0.2398 |
| C_2 | C | 2 | -0.0442 | -0.0473 | -0.0411 |
| T5_2 | T5 | 2 | 0.0417 | 0.0285 | 0.0547 |
| T10_2 | T10 | 2 | 0.0910 | 0.0757 | 0.1057 |
| T20_2 | T20 | 2 | 0.1011 | 0.0841 | 0.1174 |
| T40_2 | T40 | 2 | 0.1328 | 0.1142 | 0.1482 |
| T80_2 | T80 | 2 | 0.1784 | 0.1592 | 0.1962 |
| T160_2 | T160 | 2 | 0.2057 | 0.1871 | 0.2229 |
| T320_2 | T320 | 2 | 0.3510 | 0.3266 | 0.3724 |
| P10_1 | P10 | 1 | 0.2951 | 0.2784 | 0.3099 |
| P10_2 | P10 | 2 | 0.3046 | 0.2916 | 0.3175 |
| P320_1 | P320 | 1 | 0.0947 | 0.0861 | 0.1027 |
| P320_2 | P320 | 2 | 0.0984 | 0.0893 | 0.1077 |

Supplementary Table S4. Condition-level AP-1-specific footprint scores.

| **Condition** | **n** | **Mean** | **SD** | **Minimum** | **Maximum** | **Mean valid pairs** |
| --- | --- | --- | --- | --- | --- | --- |
| C | 2 | 0.04396 | 0.00224 | 0.04237 | 0.04554 | 55,110 |
| T5 | 2 | 0.05749 | 0.00414 | 0.05456 | 0.06041 | 55,323 |
| T10 | 2 | 0.06092 | 0.00705 | 0.05594 | 0.06590 | 55,459 |
| T20 | 2 | 0.05566 | 0.00669 | 0.05094 | 0.06039 | 55,387 |
| T40 | 2 | 0.05210 | 0.01025 | 0.04485 | 0.05935 | 55,263 |
| T80 | 2 | 0.06240 | 0.01023 | 0.05516 | 0.06963 | 55,440 |
| T160 | 2 | 0.07626 | 0.00580 | 0.07215 | 0.08036 | 55,492 |
| T320 | 2 | 0.09253 | 0.02265 | 0.07651 | 0.10855 | 55,483 |

Supplementary Table S5. Footprint dose-trend inference, replicate-specific slopes and leave-one-dose-out sensitivity.

| **Analysis** | **Subset** | **Slope per dose doubling** | **CI low** | **CI high** | **P value** |
| --- | --- | --- | --- | --- | --- |
| Pooled OLS | All doses | 0.005091 | 0.002002 | 0.008181 | 0.00398 |
| Pooled HC3 | All doses | 0.005091 | 0.000809 | 0.009374 | 0.02398 |
| Exact permutation | All doses | 0.005091 | NA | NA | 0.002109 |
| Replicate 1 | All doses | 0.003662 | -0.000749 | 0.008072 | 0.08597 |
| Replicate 2 | All doses | 0.006521 | 0.000826 | 0.012215 | 0.03212 |
| Leave-one-dose-out | Omit T5 | 0.006576 | 0.000593 | 0.012559 | 0.03463 |
| Leave-one-dose-out | Omit T10 | 0.005668 | -0.000154 | 0.011489 | 0.05513 |
| Leave-one-dose-out | Omit T20 | 0.004892 | 0.000525 | 0.009259 | 0.03201 |
| Leave-one-dose-out | Omit T40 | 0.005091 | 0.001118 | 0.009064 | 0.01763 |
| Leave-one-dose-out | Omit T80 | 0.005440 | 0.000967 | 0.009913 | 0.02242 |
| Leave-one-dose-out | Omit T160 | 0.005017 | -0.001370 | 0.011405 | 0.10931 |
| Leave-one-dose-out | Omit T320 | 0.002707 | -0.000852 | 0.006266 | 0.11943 |

Supplementary Table S6. Top 25 exploratory candidate genes ranked by repeated AP-1-rich peak assignment.

| **Gene** | **Assigned peaks** | **Maximum AP-1 occurrences** | **Minimum absolute TSS distance (bp)** | **Median baseline accessibility** | **Median T320 log2 fold change** |
| --- | --- | --- | --- | --- | --- |
| SACM1L | 4 | 5 | 20,885 | 4.595 | 1.133 |
| TRIO | 4 | 4 | 24,663 | 4.742 | 1.416 |
| CCN2 | 4 | 2 | 3,397 | 4.767 | 1.177 |
| SEMA3C | 3 | 4 | 21,455 | 2.944 | 1.424 |
| DNMBP | 3 | 3 | 3,771 | 4.190 | 1.813 |
| ADTRP | 3 | 3 | 34,484 | 3.932 | 1.794 |
| MAP3K14 | 3 | 3 | 3,782 | 4.431 | 1.571 |
| ANXA5 | 3 | 3 | 6,539 | 4.600 | 1.428 |
| GARRE1 | 3 | 3 | 20,339 | 4.963 | 1.304 |
| PLOD2 | 3 | 3 | 3,256 | 4.382 | 1.293 |
| TENM3 | 3 | 3 | 17,298 | 4.580 | 1.284 |
| CYP24A1 | 3 | 3 | 33,247 | 5.115 | 1.277 |
| COL9A1 | 3 | 3 | 9,222 | 2.970 | 1.145 |
| PALM2AKAP2 | 3 | 2 | 28,741 | 3.466 | 1.645 |
| TOP1 | 3 | 2 | 18,721 | 4.533 | 1.269 |
| ENSG00000285446 | 3 | 2 | 5,878 | 4.640 | 1.128 |
| KCNE1 | 3 | 2 | 14,383 | 4.605 | 0.871 |
| NEDD4 | 2 | 4 | 76,466 | 3.675 | 1.811 |
| ASB5 | 2 | 4 | 6,554 | 4.155 | 1.781 |
| FAM83C | 2 | 4 | 13,487 | 5.189 | 1.720 |
| ZNF331 | 2 | 4 | 18,457 | 5.599 | 1.569 |
| ITGBL1 | 2 | 4 | 2,143 | 3.090 | 1.494 |
| MBOAT4 | 2 | 4 | 2,896 | 3.434 | 1.455 |
| PGRMC2 | 2 | 4 | 65,817 | 5.604 | 1.314 |
| FHOD3 | 2 | 4 | 12,139 | 3.889 | 1.037 |

Supplementary Table S7. Representative recurring functional themes from background-corrected GO Biological Process, Reactome and KEGG ranking. The themes are presented qualitatively for hypothesis generation; no pathway-level significance or causal activation is inferred.

| **Library** | **Representative recurring themes** | **Interpretation** |
| --- | --- | --- |
| GO Biological Process | Oxidative-stress responses; phosphoinositide signalling; extracellular-matrix and MAPK regulation; protein localisation | Descriptive, hypothesis-generating context only |
| Reactome | GPCR/G-protein and PLC signalling; RHO-family GTPase cycles; CaMK-CREB and calcium-associated signalling | Descriptive, hypothesis-generating context only |
| KEGG Human | Calcium and TRP-channel regulation; AGE-RAGE/inflammatory context; Ras and HIF-associated signalling | Descriptive, hypothesis-generating context only |

Supplementary Table S8. Top 25 STRING candidate-gene hubs by weighted degree.

| **Gene** | **Weighted degree** | **Network edges** | **Assigned candidate peaks** |
| --- | --- | --- | --- |
| HIF1A | 15.312 | 26 | 1 |
| PIK3R1 | 14.297 | 21 | 1 |
| HSP90AB1 | 13.301 | 19 | 1 |
| SMAD3 | 12.013 | 19 | 1 |
| ITGB1 | 11.568 | 21 | 1 |
| TGFBR2 | 8.193 | 13 | 1 |
| VIM | 7.238 | 13 | 1 |
| POSTN | 7.132 | 14 | 1 |
| IKBKG | 6.896 | 9 | 1 |
| CCN2 | 6.739 | 11 | 4 |
| TNFSF11 | 6.528 | 12 | 1 |
| ANXA1 | 6.281 | 11 | 2 |
| ANXA5 | 6.267 | 12 | 3 |
| COL5A2 | 6.063 | 11 | 1 |
| TBK1 | 5.414 | 8 | 1 |
| YWHAQ | 5.287 | 9 | 1 |
| SNX9 | 5.095 | 8 | 1 |
| CSF1R | 4.962 | 7 | 1 |
| KAT2B | 4.894 | 6 | 1 |
| TEK | 4.780 | 8 | 1 |
| BAG3 | 4.398 | 8 | 1 |
| COMP | 4.169 | 7 | 2 |
| PLCB4 | 4.019 | 6 | 1 |
| PPIB | 3.765 | 7 | 1 |
| PLOD2 | 3.577 | 6 | 3 |

# Supplementary data and code availability

The Python analysis scripts, dataset-accession metadata, the 899-gene candidate and 4,402-gene background lists, and compact machine-readable result tables supporting the reported analyses are openly available through Zenodo, record 21520054 (doi:10.5281/zenodo.21520054). Public raw sequencing data remain available from the source repositories and are not duplicated in the release.
